# Neural Mechanisms Underlying the Impact of Psychological Resilience on Psychosocial Stress Responses

**DOI:** 10.1155/2024/5526584

**Published:** 2024-10-16

**Authors:** Danying Zhang, Xin Wang, Xiaoqiang Sun, Shulin Fang, Ge Xiong, Chang Cheng, Meiling Gu, Shuqiao Yao, Daifeng Dong, Xiang Wang

**Affiliations:** ^1^Medical Psychological Center, The Second Xiangya Hospital of Central South University, Changsha, Hunan, China; ^2^China National Clinical Research Center for Mental Disorders (Xiangya), Changsha, Hunan, China; ^3^Department of Psychology, The First Affiliated Hospital, Fujian Medical University, Fuzhou, China; ^4^Department of Psychology, National Regional Medical Center, Binhai Campus of the First Affiliated Hospital, Fujian Medical University, Fuzhou, China

**Keywords:** anxiety, cortisol, depression, fMRI, resilience, stress

## Abstract

**Background:** High psychological resilience (HR) could protect individuals from psychosocial stress and thereby make individuals less vulnerable to depression and anxiety; however, the underlying neural mechanism remains to be investigated.

**Methods:** The Montreal Imaging Stress Task (MIST) was administered to participants of 59 healthy individuals with HR and 56 individuals with low psychological resilience (LR) during functional magnetic resonance imaging (fMRI) scanning. Cortisol concentrations and subjective stress levels were collected across the MIST. Repeated measures analyses of variance were conducted to measure the group differences in subjective and cortisol stress responses. Two-sample *t*-tests were conducted to detect the group differences in stress-related brain activation and functional connectivity (FC).

**Results:** The LR group exhibited an increase in cortisol concentration after the MIST, whereas the HR group exhibited a decrease in cortisol concentration after the MIST. The LR group exhibited higher activation in the left anterior insula and lower FC between the left orbitofrontal cortex (OFC) and the right temporal pole (TP) (all *p*_*FWE*_ < 0.05). Mediation analyses revealed that the left anterior insula mediates the relationship between psychological resilience and depression and the left OFC–right TP FC mediates the relationship between psychological resilience and anxiety.

**Conclusions:** Findings highlight that the anterior insula and OFC–TP FC could be the critical neural mechanism underlying the interaction between psychological resilience and psychosocial stress. Moreover, higher anterior insula activation and lower OFC–TP FC could be the crucial neural mechanism of individuals with low psychological resilience developing into depression/anxiety when experiencing daily psychosocial stressors.

## 1. Introduction

Stress, particularly psychosocial stress, is a well-known risk factor for developing depression and anxiety in healthy individuals [1]. The effect; Mazure, Husky, and Pietrzak [2] of psychosocial stress on depression and anxiety is moderated by psychological factors, with resilience—defined as the capacity to cope with and adapt to adverse situations and recover from stress [3]—helping individuals mitigate the impact of stressful life events and prevent stress-related psychological disorders, such as depression, anxiety, and posttraumatic stress disorder (PTSD) [4–7]. Prior findings proved that individuals with high resilience (HR) could recover more quickly from stress from both the psychological and physiological perspectives [8, 9]. Also, emerging longitudinal findings suggested that individuals with higher resilience were less vulnerable to future depression and anxiety [10, 11]. Overall, prior findings highlighted the critical role of resilience in moderating the effects of stress and further reducing anxiety and depression vulnerability. Hence, investigating the neural mechanism underlying the effect of resilience on psychosocial stress responses could provide key insights into the psychopathology of depression and anxiety.

Emerging neuroimaging findings suggested that resilience is closely related to brain activity in the limbic–prefrontal circuit. Specifically, prior resting-state functional magnetic resonance imaging (fMRI) findings revealed that higher psychological resilience is associated with low fractional amplitude of low-frequency fluctuations (fALFF) in the bilateral superior frontal gyrus (SFG), right orbitofrontal cortex (OFC), and left inferior temporal gyrus (ITG) [12], higher functional connectivity (FC) between the insula and parahippocampus [13], higher FC between the OFC and superior frontal cortex [13], and lower cross-network FC between default mode network and salience network (SN) core nodes (dorsal anterior cingulate cortex and insula) [14]. Prior task-fMRI findings revealed that higher resilience is correlated with lower insula activation during both the continuous performance task for emotion detection [15, 16] and the threat processing task [17]. Additionally, Holz reviewed prior neuroimaging studies involving resilience-related factors (e.g., positive coping style and optimism) and also found a convergence in the anterior cingulate cortex, medial prefrontal cortex, OFC, and limbic regions [18]. Although prior studies highlighted that psychological resilience is closely associated with the neural activity of the limbic–prefrontal circuit, the potential effect of resilience on neural stress responses remains scarce.

The standardized paradigm suitable for fMRI (e.g., Montreal Imaging Stress Task [MIST]) has been developed and widely used in prior studies [19–23], which provide an effective protocol for investigating the potential effect of psychological resilience on stress-related neural changes (i.e., saliva concentration and brain activity). Also, the conjunction between the imaging stress paradigm and saliva cortisol is beneficial to investigate individual differences in terms of poststress recovery which is a critical feature related to resilience as we have mentioned before. Hence, in the current study, with the aim to explore the potential effect of psychological resilience on neural stress responses, we administrated a reliable imaging stress paradigm—MIST—to induce psychosocial stress. Saliva cortisol and imaging scans were collected across the MIST. The saliva concentration changes and neural brain activity (i.e., brain activation and functional coupling) were evaluated and compared in individuals with high and low psychological resilience. In light of the prior findings, we hypothesized that individuals with HR could reveal less stress-induced neural activity and faster recovery from stress in comparison to individuals with low resilience (LR), and the above HR-related neural activity could be negatively related to individuals' depressive and anxiety symptoms.

## 2. Methods

### 2.1. Participants

The healthy participants for this research were selected from a group of 1493 college students who filled out the Connor–Davidson Resilience Scale (CD-RISC), and individuals were classified as HR (score in the upper 27%; *n* = 403) or LR (score in the lower 27%; *n* = 403). Participants who agreed to undergo further data collection were given self-administered questionnaires and Structured Clinical Interview for DSM-IV-TR Axis I Disorder [24] (SCID-I) (based on DSM-IV) by professionally trained examiners; we excluded participants with current or past depressive disorders and other psychiatric disorders (see Supporting Methods for detailed eligibility criteria). All participants were informed of the experimental content and purpose and signed informed consent forms. Finally, a total of 129 participants were included in the study, with 65 subjects in the HR group (CD-RISC score 69–94 points; mean ± SD 76.08 ± 7.50) and 64 subjects in the LR group (CD-RISC score 24–56 points; mean ± SD 48.40 ± 7.78).

### 2.2. Psychological Measures

The CD-RISC scale has been described as a reliable and valid measurement of resilience [25]. This scale has good psychometric properties in a variety of samples and can distinguish individuals with HR and LR [3], with a higher total score representing higher psychological resilience. The State–Trait Anxiety Inventory (STAI) [26] and the Beck Depression Inventory (BDI) [27] were used to measure the anxiety and depressive symptoms. The Cognitive Emotion Regulation Questionnaire (CERQ) was used to assess the cognitive emotion regulation strategies that individuals employ under stress or while facing adverse events [28].

### 2.3. MIST

The MIST, which mainly induces psychosocial stress involving uncontrollability and social evaluative threat, is suitable for use in fMRI [29]. MIST has been validated to effectively induce individual stress responses and is widely used in prior fMRI studies [19, 21–23]. In the current study, MIST was designed with a block design with three 7-min fMRI sessions. Each session contains three conditions (rest, control, and stress) in this order: rest–control–stress–rest–control–stress. In the rest conditions, no task requirements were needed for the participants. In the control condition, the participants were asked to answer arithmetic questions accurately without a time limit. In the stress condition, the participants were asked to answer arithmetic questions with a time limit displayed by a visible time bar which was adapted to each participant's performance to maintain a 50% correct rate. Additionally, a performance bar is set at the top of the screen to induce evaluative stress; the performance of participants is artificially set to 80% which means that the participant's performance is “below the average subject.” Lastly, “correct” and “incorrect” feedback was given after answering the questions, and scripted verbal feedback was given after session 1 and session 2. For more details, see Figure S1.

### 2.4. Subjective and Cortisol Stress Response Measurement

The subjective stress level was assessed using a visual analog scale (VAS), with 0 corresponding to no stress and 10 being the greatest stress experienced. Participants were asked about their subjective stress levels before and after the MIST. Subjective stress responses were calculated by subtracting pre-MIST subjective stress level from post-MIST subjective stress level.

Eight salivary cortisol samples were collected (Salivette saliva collection tubes, Sarstedt, Germany) across the MIST to measure changes in cortisol levels. The acquisition time points for each participant were as follows: upon first arriving in the laboratory (Cort1), after a 30-min rest (Cort2), upon entering the MRI scanner (Cort3), after anatomical and rest scanning (Cort4), after each MIST session (Cort5–7), and after MIST for 30 min (Cort8). A human cortisol ELISA kit was used to measure the cortisol concentration in saliva samples. Cortisol increases from Cort4 to Cort7 were used to measure the cortisol changes induced by MIST; cortisol changes from Cort7 to Cort8 were used to measure the cortisol changes in the stress recovery phase.

### 2.5. fMRI Data Acquisition and Preprocessing

Scanning was conducted in a 3.0-T Siemens Magnetom Skyra scanner (Siemens Healthineers, Erlangen, Germany). Blood-oxygen-level-dependent data were acquired with an echoplanar imaging sequence with the following parameters: time to repeat = 2 s; echo time = 30 ms; flip angle = 80°; field of view = 256 × 256 mm^2^; 64 × 64 matrix; 32 slices; and thickness/gap = 4/1 mm.

Images were preprocessed using CONN19 (https://web.conn-toolbox.org/home [30]) with the following several steps: (a) Image Format Conversion, convert the original DICOM data format to the NIfTI data format; (b) slice timing correction, perform slice timing correction on the images to adjust the acquisition time to the middle slice of the scanning period; (c) realignment, estimate head motion parameters to control data noise, including translations and rotations in three directions, correct the images accordingly, and exclude subjects with translations exceeding 2 mm or rotations exceeding 2° in any direction; (d) normalization, match the subject's brain images to their T1 structural images, which are then matched to a standard template, and resample the image data to a voxel size of 3 × 3 × 3 mm^3^; and (e) smoothing, apply Gaussian smoothing to the normalized data to reduce spatial noise, with a full width at half maximum (FWHM) set to 6 mm.

### 2.6. Statistical Analysis

#### 2.6.1. Psychological and Physiological Data

Independent samples *t*-tests were used to compare group differences in general demographic data and behavioral data. Repeated measures analyses of variance were used to measure group differences in terms of subjective stress and cortisol stress responses over time.

#### 2.6.2. fMRI Data Analyses

##### 2.6.2.1. Brain Activation Analyses

Brain activation analyses were conducted using Statistical Parametric Mapping software. For the first-level analysis, a general linear model including rest, control, and stress conditions was conducted to model the stress effect for each subject. The contrast image (stress vs. control) of each subject was then submitted to the second-level analyses. Voxel-wise whole-brain analyses were conducted to detect the group differences in stress-induced brain activations between groups with HR and LR. The fMRI activation results were corrected using cluster-level family-wise error (FWE) rate correction of *p* < 0.05 surpassing an initial *p* < 0.001 voxel threshold.

##### 2.6.2.2. Generalized Psychophysiological Interaction (gPPI) Analyses

Psychophysiological interaction (PPI) analyses were performed using CONN19 (https://web.conn-toolbox.org/home). gPPI analyses were used to explore the group differences in stress-induced FC changes (stress vs. control) [31]. With regard to regions of interest (ROIs) selection, the regions exhibiting (i.e., left anterior insula) significant group differences were selected as ROIs. Additionally, since we were also interested in the effect of resilience on the FC of critical regions implicated in the stress processing, the brain regions were significantly activated (i.e., insula, thalamus, middle frontal gyrus [MFG], middle occipital gyrus [MOG], and supramarginal gyrus [SMG]) or deactivated (i.e., putamen, OFC, precuneus, angular gyrus, and rolandic operculum) by the MIST and were also selected as ROIs. The peak coordinate of the ROI was used to define a sphere with a 6 mm radius that was used as a seed for the FC analysis. During CONN's standard denoising step, potential confounding effects (white matter and cerebrospinal fluid [CSF], motion parameters, and hemodynamic response function [HRF]-convolved task condition effects) were linear regressed. We next extracted time series for our gPPI analysis from the first-level general linear models for each participant: (1) extracting HRF-convolved psychological time series variables for task condition (stress vs. control); (2) extracting time series of seed regions; and (3) generating PPI interaction terms between ROI signal and experimental design. The effect of the interaction term was detected and then submitted to the group-level analysis. The gPPI results were corrected using cluster-level FWE rate correction of *p* < 0.05 surpassing an initial *p* < 0.001 voxel threshold.

#### 2.6.3. Correlation and Mediation Analyses

Partial correlation analyses, controlling for age, sex, and years of education, were performed among brain regions/FCs exhibiting significant group differences and subjective and cortisol stress responses. Additionally, partial correlations between the brain regions/FCs exhibiting significant group differences and behavioral measures (CD-RISC score, BDI score, STAI score, and CERQ score) were also tested and also tested the mediation role of brain regions/FCs exhibiting significant group differences between resilience and depressive as well as anxiety symptoms. Mediation analyses were conducted using the bootstrap method [32, 33] of the process3.4 plugin built in Statistical Package for the Social Sciences (SPSS) 21.0.

## 3. Results

### 3.1. Sample Characteristics

After excluding participants with excessive head movements (6 HR, 8 LR) during MIST task scanning, 59 participants with HR and 56 participants with LR were left for analysis. Demographic and behavioral characteristics of the HR and LR group were summarized in Table 1. The HR group exhibited significantly higher CD-RISC scores as well as its subscale scores than the LR group (all *p* < 0.05). Additionally, the HR group exhibited higher scores in several CERQ subscales (i.e., positive refocus, refocus on planning, and positive reappraisal) but lower scores in the CERQ-catastrophizing subscale in comparison to the LR group (all *p* < 0.05). In terms of depression and anxiety, the HR group scored lower than the LR group (all *p* < 0.05) on BDI, SAI, and TAI scores (all *p* < 0.05).

### 3.2. Subjective Stress Response

**Figure 1:**
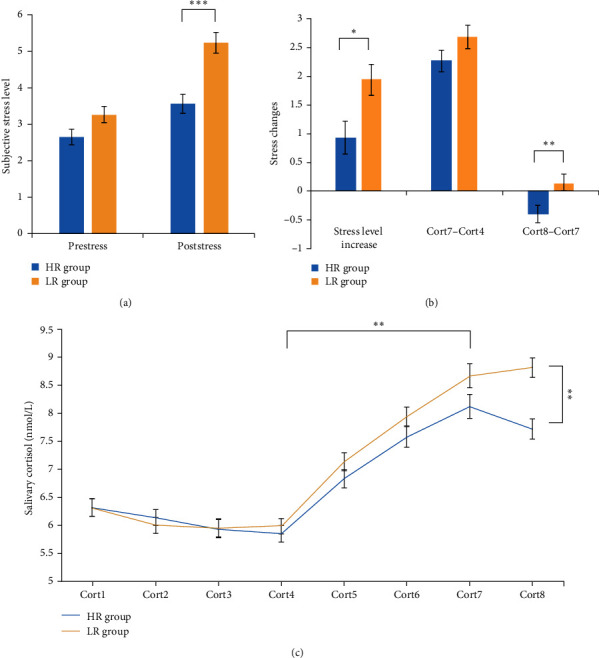
Physiological and psychological responses to psychosocial stress: (a) group differences in subjective stress levels before and after stress; (b) group differences in terms of subjective stress changes and cortisol stress changes during (Cort7–Cort4) and after (Cort8–Cort7) the stress; and (c) group differences in the cortisol concentration across the Montreal Imaging Stress Task. HR, high resilience; LR, low resilience. *⁣*^*∗*^*p* < 0.05, *⁣*^*∗∗*^*p* < 0.01, *⁣*^*∗∗∗*^*p* < 0.001.

Repeated measures analysis of variance (ANOVA) on subjective stress levels showed a significant effect of *time* (post-MIST > pre-MIST; *F*_1113_ = 52.51, *p* < 0.001; Figure 1a), group (HR < LR; *F*_1113_ = 12.77, *p* < 0.001), and time × group interaction (*F*_1113_ = 14.77, *p*=0.012). Further, simple-effect analysis revealed that the LR group exhibited significantly higher post-MIST subjective stress levels in comparison to the HR group (*p* < 0.001; Figure 1a); no other significant effects emerged (all *p* > 0.05). In terms of the subjective stress responses (post-MIST minus pre-MIST), the LR group exhibited significantly higher subjective stress responses in comparison to the HR group (*t* = −2.56, *p*=0.012; Figure 1b).

### 3.3. Cortisol Stress Response

Repeated measures ANOVA on pre-MIST cortisol concentration (Cort1–Cort4) revealed a significant main effect of time (*F*_3339_ = 4.41, *p*=0.008; Figure 1c), but the main effect of *group* (*F*_1113_ < 0.01, *p*=0.950) and the *time* × *group* interaction effect (*F*_3339_ = 0.36, *p*=0.746) were not significant. Repeated measures ANOVA test on cortisol concentration during and after the MIST (Cort4–Cort7) showed a significant main effect of time (Cort7 > Cort4; *F*_3339_ = 193.31, *p* < 0.001; Figure 1c); no significant effects of group (*F*_1113_ = 2.56, *p*=0.112) and time × group interaction (*F*_3339_ = 1.25, *p*=0.293) emerged. Repeated measure ANOVA test on cortisol concentration after the MIST (Cort7–Cort8) revealed a significant main effect of *group* (HR < LR; *F*_1113_ = 10.45, *p*=0.002; Figure 1c) and time × group (*F*_1113_ = 6.90, *p*=0.01) interaction; no significant main effect of time emerged (*F*_1113_ = 1.61, *p*=0.207). Further, simple-effect analysis revealed that the LR group exhibited significantly higher cortisol concentrations in comparison to the HR group in Cort8 (*p*=0.002; Figure 1c); no other significant effects emerged (all *p* > 0.05).

Independent samples *t*-tests on cortisol stress changes (Cort7–Cort4) revealed no significant group differences (*t* = −1.51, *p*=0.133; Figure 1b). Independent samples *t*-tests on cortisol stress recovery-related changes (Cort8–Cort7) revealed that the LR group exhibited higher cortisol changes (*t* = −2.63, *p*=0.010; Figure 1b) in comparison to the HR group.

### 3.4. Stress-Related Brain Activation and Connectivity

As revealed by whole-brain voxel-wise one-sample *t*-tests, the MIST significantly activated the insula, thalamus, MFG, MOG, and SMG and significantly deactivated the putamen, precuneus, OFC, angular gyrus, Rolandic operculum (*p*_*FWE*_ < 0.001; Figure S2 and Table S1). Brain activation patterns of the HR and LR group were also shown in Figure S2 and Tables S2 and S3.

**Figure 2:**
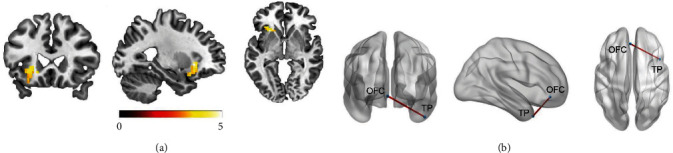
Group differences in stress-related brain activation and functional connectivity: (a) group differences in the left insula during psychosocial stress processing and (b) group differences in the functional connectivity between the orbitofrontal cortex and the temporal pole during stress processing. The color bar represents the *t*-value range. The red line indicates a significant increase in functional connectivity between the two brain regions. OFC, orbitofrontal cortex; TP, temporal pole.

A two-sample *t*-test between the HR and LR group in terms of brain activation revealed that the LR group exhibited significantly higher activation in the left anterior insula in comparison to the HR group (*p*_FWE_ < 0.05; Figure 2a and Table S4). A two-sample *t*-test between the HR and LR group in terms of the FCs revealed that the LR group exhibited significantly lower left OFC–temporal pole (TP) connectivity in comparison to the HR group (*p*_FWE_ < 0.05; Figure 2b). The brain functional connection patterns of the whole group, HR and LR, were also shown in Figures S3, S4, and S5.

### 3.5. Correlation and Mediation Analysis

#### 3.5.1. Results of Partial Correlation Analysis

After controlling for age, sex, and years of education, the activity of the left insula was negatively correlated with CS-RISC score (*r* = −0.41, *p* < 0.001, *p*_FDR_ < 0.001) and positively correlated with BDI score (*r* = 0.35, *p*=0.002, *p*_FDR_ = 0.001), SAI score (*r* = 0.19, *p*=0.047, *p*_FDR_ = 0.093), Cort7–Cort4 (*r* = 0.25, *p*=0.007, *p*_FDR_ = 0.024), and Cort8–Cort7 (*r* = 0.20, *p*=0.036, *p*_FDR_ = 0.091). The left OFC–TP FC was positively correlated with the CD-RISC score (*r* = 0.35, *p* < 0.001, *p*_FDR_ = 0.002), CERQ-positive reappraisal score (*r* = 0.29, *p*=0.002, *p*_FDR_ = 0.006), and CERQ self-blame score (*r* = 0.21, *p*=0.027, *p*_FDR_ = 0.069) and negatively correlated with SAI score (*r* = −0.33, *p* < 0.001, *p*_FDR_ = 0.002). No other significant effects were detected between the left insula/left OFC–TP FC and behavioral measures (all *p* > 0.05). See Table S5 for more details.

#### 3.5.2. Results of Mediation Analysis

**Figure 3:**
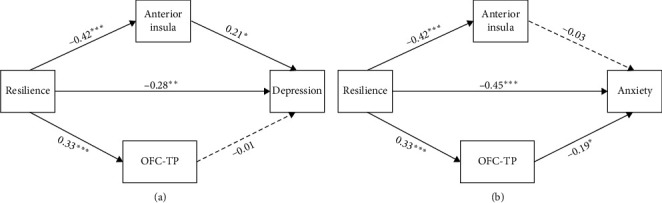
Mediation role of the anterior insula and OFC–TP FC between resilience and depression/anxiety. (a) Parallel mediation model for resilience and depression via the anterior insula and OFC–TP FC. (b) Parallel mediation model for resilience and anxiety via the anterior insula and OFC–TP FC. OFC, orbitofrontal cortex; TP, temporal pole. *⁣*^*∗*^*p* < 0.05, *⁣*^*∗∗*^*p* < 0.01, *⁣*^*∗∗∗*^*p* < 0.001.

Parallel mediation analysis revealed that the total effect of resilience on depression (*β* = −0.28, *p*=0.005, 95% CI [−0.197,−0.535]; Figure 3a) and anxiety symptoms (*β* = −0.45, *p* < 0.001, 95% CI [−0.338,−0.654]; Figure 3b) was significant. Additionally, the mediation effect of the left anterior insula between the resilience and depressive symptoms was significant (95% CI [−0.011, −0.180]; Figure 3a); the mediation effect of left OFC–TP FC between the resilience and anxiety symptoms was significant (95% CI [−0.012,−131]; Figure 3b). See Tables S6–S9 for more details.

## 4. Discussion

The overarching goal of the current study was to investigate the impact of psychological resilience on cortisol and brain activity changes induced by psychosocial stressors. Our cortisol findings revealed that the HR group showed a decrease in cortisol concentration after the MIST; in contrast, the LR group still exhibited an increase in cortisol concentration after the MIST. Our fMRI findings revealed that the LR group exhibited higher activation in the insula and lower left OFC–TP FC during psychosocial processing relative to the HR group; further mediation analyses confirmed that the insula and OFC–TP FC play a critical role in mediating the psychological resilience and anxiety/depression. Collectively, these findings provide insights into the potential neural mechanism underlying the protective effect of psychological resilience for depression/anxiety from psychosocial stress.

Consistent with our hypothesis, our findings revealed that the HR group recovered from the psychosocial stress faster relative to the LR group as shown by the cortisol concentration changes after the MIST. These findings highlighted the protective role of psychological resilience from psychosocial stress, and this protective effect was more pronounced in the speed of returning to a stable state after the onset of stress. In support of these findings, prior literature also found that individuals with HR exhibited a higher subjective stress response [9] and a faster subjective and physiological (heart rate, skin conductance, etc.) stress recovery [9, 34] relative to the individuals with LR. Collectively, these prior findings support that psychological resilience plays a protective role from acute stress from both the psychological and physiological perspectives.

The current fMRI findings revealed that the LR group exhibited higher anterior insula activation relative to the HR group during stress processing, highlighting the critical role of anterior insula in the effect of psychological resilience on stress processing. Consistently, prior rest-fMRI findings revealed that the regional homogeneity of the anterior insula and anterior cingulate cortex could predict individual differences in psychological resilience [12]. Also, higher anterior insula activation was observed in the LR individuals in aversive interoceptive stimulus processing and emotion detection [15, 16]. The anterior insula, as a key hub of the SN, is involved with integrating physiological states, emotions, and cognition and interacts with other intrinsic networks such as the default mode network and central executive network [35]. Mounting evidence suggests that the anterior insula is frequently activated when individuals focus on internal states [35, 36]. Along this line, the higher stress-related insula activation in the LR group could reveal a heightened sensitivity to interoceptive information in stress processing; the positive correlation between insula activation and cortisol stress responses observed in the current study further supports this speculation. Additionally, our mediation analyses also found the activation of the anterior insula plays a critical mediating role between psychological resilience and depression, which further suggested the higher stress-related insula activation in the LR group could lead to depression when experiencing daily stressors.

With regard to the stress-induced FC changes, our fMRI findings revealed that the LR group exhibited lower OFC–TP FC in comparison to the HR group. The critical role of OFC-related FCs in resilience has been previously reported by prior studies. Shi et al. [13] found that the OFC–SFG resting-FC is positively correlated with psychological resilience; moreover, the OFC–SFG FC is also closely associated with flexible emotional resources utilization and flexible controllability when processing affective information. The TP, as reviewed by a prior study [37], is mainly involved in recognizing the significance of social signals and transferring the highly processed perceptual inputs with internal emotional responses. Along this line, the lower stress-related OFC–TP FC in the LR group could reveal a weakened ability in emotion processing and behavioral controllability when processing psychosocial stress. Consistently, the current study also found lower OFC–TP FC is correlated with maladaptive emotion regulation strategies. Additionally, our mediation analyses found that the OFC–TP FC mediates the effects of psychological resilience on anxiety, which further suggested that the lower stress-related OFC–TP FC in the LR group could lead to higher anxiety emotion or anxiety disorders when experiencing daily stressors.

Several limitations of the current study should be mentioned. First, our sample mainly included the youth aged 18–26; further studies were warranted to test whether the current findings could be generalized to individuals of other age groups. Second, in the current study using a cross-sectional design, the mediation role of the anterior insula and OFC–TP between psychological resilience and depression/anxiety should be validated by a longitudinal design. Third, the participants were not instructed to brush their teeth before salivary sample collection and not eat/drink before the fMRI scanning, which may affect the measurement of cortisol concentration. Fourth, the current study did not measure and control the adverse childhood experiences which could affect neural psychosocial stress responses. Fifth, symptoms related to PTSD should be measured and considered in future related studies, which could provide additional insights into understanding the relationship among stress, resilience, and stress-related disorders. Finally, to exclude the effect of current and past diagnoses of psychiatric disorders on neural psychosocial stress responses, only healthy individuals with no history of psychiatric disorders were included in the current study; further verification of our findings in individuals with psychiatric disorders, particularly for stress-related disorders, is warranted.

In spite of these limitations, the current study unraveled the potential effect of psychological resilience on cortisol stress recovery and anterior insula and OFC-TP FC during stress processing, and further confirmed the mediating role of stress-related anterior insula and OFC-TP FC between psychological resilience and depression/anxiety. Our findings revealed that higher psychological resilience plays a protective role from psychosocial stress, mainly through a faster cortisol stress recovery, a lower insula activation as well as a higher OFC-TP FC; furthermore, the lower insula activation and higher OFC-TP FC observed in the HR group could reveal decreased stress perception and higher stress regulation, which may thereby lessen the daily stressor accumulation and protected individuals from anxiety and depression.

## Figures and Tables

**Table 1:** Demographic and behavioral characteristics.

Characteristics	HR (*N* = 59)	LR (*N* = 56)	*χ* ^2^/*t*	*p*	Cohen's *d*
	Mean ± SD	Mean ± SD			
Sex (male/female)	28/31	25/31	0.09	0.762	—
Age (year)	21.10 ± 2.06	20.48 ± 1.50	1.84	0.069	—
Education (year)	14.76 ± 1.59	14.50 ± 1.41	0.93	0.352	—
CD-RISC	76.19 ± 7.62	49.63 ± 6.96	19.49	<0.001	3.64
BDI	2.86 ± 3.83	6.19 ± 4.75	−4.12	<0.001	0.77
SAI	32.53 ± 7.00	40.54 ± 7.43	−5.96	<0.001	1.11
TAI	36.45 ± 7.01	45.61 ± 5.66	−7.63	<0.001	−1.44
CERQ					
Self-blame	12.69 ± 1.61	12.46 ± 1.54	0.79	0.434	—
Acceptance	14.55 ± 2.49	13.84 ± 2.10	1.66	0.100	—
Rumination	11.75 ± 3.72	12.43 ± 2.24	−1.20	0.233	—
Positive refocus	12.88 ± 2.75	11.77 ± 2.24	2.37	0.019	0.44
Refocus on planning	16.51 ± 2.45	13.63 ± 2.51	6.23	<0.001	1.16
Positive reappraisal	16.86 ± 2.60	12.85 ± 2.34	8.68	<0.001	1.62
Putting into perspective	11.02 ± 2.96	10.63 ± 2.07	0.83	0.410	—
Catastrophizing	7.07 ± 2.52	8.77 ± 2.57	−3.59	<0.001	0.67
Blaming others	9.18 ± 3.13	9.99 ± 2.00	−1.64	0.101	—

Abbreviations: BDI, Beck Depression Inventory; CD-RISC, Connor–Davidson Resilience Scale; CERQ, Cognitive Emotion Regulation Questionnaire; HR, high resilience; LR, low resilience; SAI, State Anxiety Inventory; TAI, Trait Anxiety Inventory.

## Data Availability

The data that support the findings of this study are available on request from the corresponding author. The data are not publicly available due to privacy or ethical restrictions.

## Supporting Information

Supporting Information The supporting information include supporting methods (participants, data acquisition, data analysis, and mediation analysis) and supporting results (Tables S1–S9 and Figures S1–S5). Table S1. Stress-induced brain activation pattern in the whole group. Table S2. Stress-induced brain activation pattern in the HR group. Table S3. Stress-induced brain activation pattern in the low resilience group. Table S4. Group differences in stress-related brain activation. Table S5. Partial correlation analysis of brain activation and functional connectivity results with other behavioral variables controlling age, sex, and years of education. Table S6. The result of the parallel mediation model using depressive symptoms as the dependent variable. Table S7. Paths and effect size for parallel mediation model using depressive symptoms as the dependent variable. Table S8. The result of the parallel mediation model using anxiety symptoms as the dependent variable. Table S9. Paths and effect size for parallel mediation model using anxiety symptoms as the dependent variable. Figure S1. An overview of stressful tasks. Figure S2. Brain functional activation/deactivation induced by stressful tasks. Figure S3. The brain functional connection patterns of the whole group under stress. Figure S4. Brain functional connectivity patterns of the high resilience group. Figure S5. Brain functional connectivity patterns of low resilience group.

## References

[1] Hammen C. (2005). Stress and Depression. *Annual Review of Clinical Psychology*.

[2] Mazure C. M., Husky M. M., Pietrzak R. H. (2023). Stress as a Risk Factor for Mental Disorders in a Gendered Environment. *JAMA Psychiatry*.

[3] Connor K. M., Davidson J. R. T. (2003). Development of a New Resilience Scale: The Connor-Davidson Resilience Scale (CD-RISC). *Depression and Anxiety*.

[4] Ahmed A. S. (2007). Post-Traumatic Stress Disorder, Resilience and Vulnerability. *Advances in Psychiatric Treatment*.

[5] Faye C., Mcgowan J. C., Denny C. A., David D. J. (2018). Neurobiological Mechanisms of Stress Resilience and Implications for the Aged Population. *Current Neuropharmacology*.

[6] Thompson N. J., Fiorillo D., Rothbaum B. O., Ressler K. J., Michopoulos V. (2018). Coping Strategies as Mediators in Relation to Resilience and Posttraumatic Stress Disorder. *Journal of Affective Disorders*.

[7] Zheng K., Chu J., Zhang X. (2022). Psychological Resilience and Daily Stress Mediate the Effect of Childhood Trauma on Depression. *Child Abuse & Neglect*.

[8] Mikolajczak M., Roy E., Luminet O., de Timary P. (2008). Resilience and Hypothalamic-Pituitary-Adrenal Axis Reactivity Under Acute Stress in Young Men. *Stress*.

[9] Zuo X., Li P., Min L. (2012). Individual Subjective Tension and Salivary Levels of *α*-Amylase and Glucocorticoid in College Students with Different Levels of Resilience in Trier Social Stress Test. *Journal of Third Military Medical University*.

[10] Hiyoshi A., Udumyan R., Osika W., Bihagen E., Fall K., Montgomery S. (2015). Stress Resilience in Adolescence and Subsequent Antidepressant and Anxiolytic Medication in Middle Aged Men: Swedish Cohort Study. *Social Science & Medicine*.

[11] Navrady L. B., Adams M. J., Chan S. W. Y., McIntosh A. M. (2018). Major Depressive Disorder Working Group of the Psychiatric Genomics Consortium, Genetic Risk of Major Depressive Disorder: The Moderating and Mediating Effects of Neuroticism and Psychological Resilience on Clinical and Self-Reported Depression. *Psychological Medicine*.

[12] Kong F., Hu S., Wang X., Song Y., Liu J. (2015). Neural Correlates of the Happy Life: The Amplitude of Spontaneous Low Frequency Fluctuations Predicts Subjective Well-Being. *NeuroImage*.

[13] Shi L., Sun J., Wei D., Qiu J. (2019). Recover from the Adversity: Functional Connectivity Basis of Psychological Resilience. *Neuropsychologia*.

[14] Brunetti M., Marzetti L., Sepede G. (2017). Resilience and Cross-Network Connectivity: A Neural Model for Post-Trauma Survival. *Progress in Neuro-Psychopharmacology and Biological Psychiatry*.

[15] Paulus M. P., Flagan T., Simmons A. N. (2012). Subjecting Elite Athletes to Inspiratory Breathing Load Reveals Behavioral and Neural Signatures of Optimal Performers in Extreme Environments. *PLoS ONE*.

[16] Simmons A. N., Fitzpatrick S., Strigo I. A. (2012). Altered Insula Activation in Anticipation of Changing Emotional States: Neural Mechanisms Underlying Cognitive Flexibility in Special Operations Forces Personnel. *NeuroReport*.

[17] Waugh C. E., Wager T. D., Fredrickson B. L., Noll D. C., Taylor S. F. (2008). The Neural Correlates of Trait Resilience when Anticipating and Recovering from Threat. *Social Cognitive and Affective Neuroscience*.

[18] Holz N. E., Tost H., Meyer-Lindenberg A. (2020). Resilience and the Brain: A Key Role for Regulatory Circuits Linked to Social Stress and Support. *Molecular Psychiatry*.

[19] Dedovic K., D’Aguiar C., Pruessner J. C. (2009). What Stress Does to Your Brain: A Review of Neuroimaging Studies. *The Canadian Journal of Psychiatry*.

[20] Dong D., Ironside M., Belleau E. L. (2022). Sex-Specific Neural Responses to Acute Psychosocial Stress in Depression. *Translational Psychiatry*.

[21] Lederbogen F., Kirsch P., Haddad L. (2011). City Living and Urban Upbringing Affect Neural Social Stress Processing in Humans. *Nature*.

[22] Ming Q., Zhong X., Zhang X. (2017). State-Independent and Dependent Neural Responses to Psychosocial Stress in Current and Remitted Depression. *American Journal of Psychiatry*.

[23] Pruessner J. C., Dedovic K., Khalili-Mahani N. (2008). Deactivation of the Limbic System During Acute Psychosocial Stress: Evidence from Positron Emission Tomography and Functional Magnetic Resonance Imaging Studies. *Biological Psychiatry*.

[24] Passos I. C., Jansen K., de A. Cardoso T. (2016). Clinical Outcomes Associated with Comorbid Posttraumatic Stress Disorder among Patients with Bipolar Disorder. *The Journal of Clinical Psychiatry*.

[25] Cheng C., Dong D., He J., Zhong X., Yao S. (2020). Psychometric Properties of the 10-Item Connor-Davidson Resilience Scale (CD-RISC-10) in Chinese Undergraduates and Depressive Patients. *Journal of Affective Disorders*.

[26] Spielberger C. D., Gorsuch R. L., Lushene R. (2003). *Manual for the State-Trait Anxiety Inventory (Self-evaluation Questionnaire)*.

[27] Beck A. T., Ward C. H., Mendelson M., Mock J., Erbaugh J. (1961). An Inventory for Measuring Depression. *Archives of General Psychiatry*.

[28] Garnefski N., Kraaij V., Spinhoven P. (2001). Negative Life Events, Cognitive Emotion Regulation and Emotional Problems. *Personality and Individual Differences*.

[29] Dedovic K., Renwick R., Mahani N. K., Engert V., Lupien S. J., Pruessner J. C. (2005). The Montreal Imaging Stress Task: Using Functional Imaging to Investigate the Effects of Perceiving and Processing Psychosocial Stress in the Human Brain. *Journal of Psychiatry & Neuroscience: JPN*.

[30] Whitfield-Gabrieli S., Nieto-Castanon A. (2012). *Conn*: A Functional Connectivity Toolbox for Correlated and Anticorrelated Brain Networks. *Brain Connectivity*.

[31] Luo S., Yu D., Han S. (2016). Genetic and Neural Correlates of Romantic Relationship Satisfaction. *Social Cognitive and Affective Neuroscience*.

[32] Hayes A. F., Preacher K. J. (2010). Mediation and the Estimation of Indirect Effects in Political Communication Research. *Sourcebook for Political Communication Research*.

[33] Pandey D., Shrivastava P. (2017). Mediation Effect of Social Support on the Association Between Hardiness and Immune Response. *Asian Journal of Psychiatry*.

[34] Mao S. (2008). *Effect Mechanism Between Resiliency and Ego-Resilience*.

[35] Zhu Y., Wang Y., Yang Z., Wang L., Hu X. (2020). Endogenous Cortisol-Related Alterations of Right Anterior Insula Functional Connectivity Under Acute Stress. *Journal of Affective Disorders*.

[36] Napadow V., Lee J., Kim J. (2013). Brain Correlates of Phasic Autonomic Response to Acupuncture Stimulation: An Event-Related fMRI Study. *Human Brain Mapping*.

[37] Olson I. R., Plotzker A., Ezzyat Y. (2007). The Enigmatic Temporal Pole: A Review of Findings on Social and Emotional Processing. *Brain: A Journal of Neurology*.

