# Supplementary material for: Neural Mechanisms Underlying the Impact of Psychological Resilience on Psychosocial Stress Responses
Source: Depress Anxiety. 2024 Oct 16;2024:5526584. doi: 10.1155/2024/5526584 (PMC11919183; doi:10.1155/2024/5526584)
Supplement: Supporting Information — The supporting information include supporting methods (participants, data acquisition, data analysis, and mediation analysis) and supporting results (Tables S1–S9 and Figures S1–S5). Table S1. Stress-induced brain activation pattern in the whole group. Table S2. Stress-induced brain activation pattern in the HR group. Table S3. Stress-induced brain activation pattern in the low resilience group. Table S4. Group differences in stress-related brain activation. Table S5. Partial correlation analysis of brain activation and functional connectivity results with other behavioral variables controlling age, sex, and years of education. Table S6. The result of the parallel mediation model using depressive symptoms as the dependent variable. Table S7. Paths and effect size for parallel mediation model using depressive symptoms as the dependent variable. Table S8. The result of the parallel mediation model using anxiety symptoms as the dependent variable. Table S9. Paths and effect size for parallel mediation model using anxiety symptoms as the dependent variable. Figure S1. An overview of stressful tasks. Figure S2. Brain functional activation/deactivation induced by stressful tasks. Figure S3. The brain functional connection patterns of the whole group under stress. Figure S4. Brain functional connectivity patterns of the high resilience group. Figure S5. Brain functional connectivity patterns of low resilience group. [file 5526584.f1.pdf]

Neural mechanisms underlying the impact of psychological resilience on psychosocial stress responses

Supplemental Information

Supplemental Methods

Participants

The inclusion criteria included CD-RISC score ≥ 69 points or ≤ 56 points, college students aged 18-26, healthy body, understanding of the research content, voluntary participation and ability to complete the entire experiment, and signed informed consent. Exclusion criteria included a current or previous diagnosis of any psychiatric disorder consistent with DSM-IV-TR, a family history of psychiatric disorders or a history of central nervous system disorders (eg, head trauma, seizures, etc.), or a history of major medical illnesses, substance abuse or substance use history of dependence, contraindications to MRI (metal in the body), and other circumstances in which the scan cannot be completed.

Data Acquisition

After the participants were familiar with the experimental procedure, the professionally trained examiners conducted structured clinical interviews based on DSM-IV and psychological questionnaires which were used to obtain the general demographic data of the participants, the resilience level, commonly used cognitive emotion regulation strategies under stress or negative events, and the level of depression and anxiety in the last week.

Data analysis

fMRI data preprocessing

(1) Image format conversion: Convert the original dicom data format to the nii data format.

(2) Slice time correction: Slice time correction corrects these slice-related delays by shifting the time series of each slice to time-align all slices to a reference time point, and the reference scan time is the middle layer of the scan time.

(3) Realignment: The noise of the data is controlled by estimating head motion parameters, including translation and rotation in each of the three directions, and the image is corrected.

Subjects with a translation of more than 2 mm or a rotation of more than 2° in either direction were excluded.

(4) Normalization: The subject's brain image was matched to the subject's T1 structural image, the T1 structural image was matched to the standard template, and the image data was resampled with a voxel size of $3 \times 3 \times 3$ mm^3.

(5) Smoothing: Gaussian smoothing was performed on the normalized data to reduce spatial noise, and the full width at half maximum (FWHM) value was set to 6mm.

Mediation analysis

The bootstrap method (Hayes,A.F. & Preacher, K. J., 2010; Pandey & Shrivastava, 2017) was used to test the mediation effect. The selected model was model 4 and the number of samples was 5000. Age was included as a covariate to control for possible effects of demographic data on the results. In order to eliminate the influence of the dimension and value range differences between variables on the data results, the data were standardized before the model was constructed. The standardization method in this study was Z-score standardization (Das et al., 2020). We used Harman's single-factor test to detect common method bias in the data, and exploratory factor analysis was carried out on all items of the CD-RISC and BDI, anterior insula activation, and the functional connection strength between left OFC and right TP. We extracted 14 factors with eigenvalues > 1, of which the variance contribution rate of the first common factor was 27% (i.e., lower than the standard 50%) indicating that there was no serious common method bias effect among the variables (Podsakoff & Organ, 1986).

Table S1. Stress-induced brain activation pattern in the whole group

	**Brain region**	**Side**	**MNI coordinates**			***t***	$\boldsymbol{P_{FWE}}$
			**x**	**y**	**z**		
Activation	Insula	L	-30	21	6	7.34	<0.001
		R	33	27	6	9.30	<0.001
	Thalamus	L	-15	-21	18	7.63	<0.001
		R	15	-18	18	8.63	<0.001
	MFG	L	-33	42	33	6.50	<0.001
	MOG	R	24	-90	9	15.22	<0.001
	SMG	R	60	-36	24	7.17	<0.001
Deactivation	Putamen	L	-18	9	-9	12.21	<0.001
	OFC	L	-3	39	-9	9.78	<0.001
	Precuneus	R	3	-66	24	6.32	<0.001
	Angular	L	-51	-72	33	9.96	<0.001
		R	51	-72	33	10.08	<0.001
	ROL	R	39	-15	18	6.76	<0.001

Note: MFG: Middle frontal gyrus; MOG: Middle occipital gyrus; SMG: Supramarginal gyrus; OFC: orbitofrontal cortex; ROL: Rolandic operculum; L/R: Left/Right; FEW: Family-wise error.

Table S2. Stress-induced brain activation pattern in the high resilience group

	Brain region	Side	MNI coordinates			t	P_{FWE}
			x	y	z		
Activation	Insula	R	30	27	6	6.24	<0.001
	Thalamus	L	-12	-21	15	6.78	<0.001
		R	15	-18	18	7.64	<0.001
	SFG	R	24	-3	60	9.27	<0.001
	MOG	L	-24	-90	6	9.55	<0.001
		R	24	-90	9	11.98	<0.001
	MFG	R	33	42	30	6.05	<0.001
	Precuneus	L	-57	9	30	6.81	<0.001
	MTG	L	-48	-66	0	7.70	<0.001
	SPG	L	-21	-54	60	8.65	<0.001
Deactivation	Putamen	L	-18	12	-9	9.06	<0.001
	Angular	R	60	-63	33	7.81	<0.001
	Insula	R	48	0	-6	7.38	<0.001
	TP	R	39	21	-33	6.65	<0.001
	OFC	R	21	33	-15	6.62	<0.001
	Hippocampus	L	-18	-9	-21	6.39	<0.001
	SPG	L	-36	-69	54	7.83	<0.001

Note: MFG: Middle frontal gyrus; MOG: Middle occipital gyrus; TP: Temporal pole; SFG: Superior frontal gyrus; MTG: Middle temporal gyrus; SPG: Superior parietal gyrus; OFC: orbitofrontal cortex; L/R: Left/Right; FEW: Family-wise error.

Table S3. Stress-induced brain activation pattern in the low resilience group

	**Brain region**	**Side**	**MNI coordinates**			***t***	***P_{FWE}***
			**x**	**y**	**z**		
Activation	Insula	L	-30	24	9	7.59	<0.001
		R	33	27	3	7.73	<0.001
	MFG	R	33	54	18	7.57	<0.001
	SOG	L	-15	-96	9	10.49	<0.001
	MOG	R	39	-78	15	10.41	<0.001
	SPG	L	-21	-57	60	9.69	<0.001
	SFG	R	33	0	66	9.30	<0.001
	Precuneus	L	-27	-6	54	8.45	<0.001
	SMA	L	-3	6	54	7.89	<0.001
Deactivation	Putamen	L	-18	6	-9	8.64	<0.001
	Angular	L	-54	-69	33	6.81	<0.001
		R	51	-72	36	6.95	<0.001
	ITG	L	-39	6	-36	7.46	<0.001
	OFC	L	-3	39	-9	6.63	<0.001
	OFC	L	-24	30	-15	6.26	<0.001
	TP	R	36	18	-27	6.17	<0.001

Note: MFG: Middle frontal gyrus; MOG: Middle occipital gyrus; TP: Temporal pole; SOG: Superior occipital gyrus; SPG: Superior parietal gyrus; SFG: Superior frontal gyrus; SMA: Supplementary motor area; ITG: Inferior temporal gyrus; OFC: orbitofrontal cortex; L/R: Left/Right; FEW: Family-wise error.

Table S4 Group differences in stress-related brain activation

**Brain region**	**Side**	**MNI coordinates**			***t***	P_{FWE}
		**x**	**y**	**z**		
anterior insula	L	-27	21	-3	4.94	0.018

Note: FWE: Family-wise error.

Table S5. Partial correlation analysis of brain activation and functional connectivity results with other behavioral variables controlling age, sex, and years of education

	Anterior insula			OFC-TP		
	r	*p*	p_{FDR}	*r*	*p*	p_{FDR}
CD-RISC	-0.41	<0.001	<0.001	0.35	<0.001	0.002
BDI	0.35	0.002	0.001	-0.16	0.101	0.169
SAI	0.19	0.047	0.093	-0.33	<0.001	0.002
Refocus on planning	-0.13	0.172	0.215	0.17	0.076	0.151
Positive refocus	-0.06	0.555	0.616	-0.02	0.879	0.879
Positive reappraisal	-0.17	0.077	0.11	0.29	0.002	0.006
Catastrophizing	0.18	0.056	0.094	-0.14	0.138	0.197
Self-blame	0.04	0.664	0.664	0.21	0.027	0.069
Cort7-Cort4	0.25	0.007	0.024	-0.13	0.189	0.239
Cort8-Cort7	0.2	0.036	0.091	-0.04	0.671	0.745

Note: OFC-TP: the functional connection strength between left OFC and right TP.

Table S6. The result of the parallel mediation model using depressive symptoms as the dependent variable

	anterior insula			OFC-TP			depression		
	β	*SE*	*t*	*β*	*SE*	*t*	*β*	*SE*	*t*
Age	-0.11	0.09	-1.25	-0.17	0.09	-1.95	-0.19	0.09	-2.20^{*}
Resilience	-0.42	0.09	-4.86	0.33	0.09	3.75^{***}	-0.28	0.10	-2.86
Anterior insula							0.21	0.09	2.24^{*}
OFC-TP							-0.01	0.09	-0.11
	R^2	0.20			0.13			0.23	
	F	13.60^{***}			8.14^{***}			8.39^{***}	

Note: OFC-TP: the functional connection strength between left OFC and right TP; SE: standard error. *β:* standardized regression coefficient; $^{*}p < 0.05$, $^{**}p < 0.01$, $^{***}p < 0.001$.

Table S7 Paths and effect size for parallel mediation model using depressive symptoms as the dependent variable

	Effect size	Boot-LLCI	Boot-ULCI
Direct effect			
**resilience→depression**	**-0.276**	**-0.467**	**-0.085**
Indirect effect			
**resilience→anterior insula→depression**	**-0.087**	**-0.180**	**-0.011**
resilience→OFC-TP→depression	-0.003	-0.059	0.055
**Total effect**	**-0.366**	**-0.535**	**-0.197**

Note: Bold parts indicate significant paths; LLCI = lower level of 95% confidence interval, ULCI- upper level of 95% confidence interval. OFC-TP: the functional connection strength between left OFC and right TP;

Table S8. The result of the parallel mediation model using anxiety symptoms as the dependent variable

	anterior insula			OFC-TP			anxiety		
	β	*SE*	*t*	*β*	*SE*	*t*	*β*	*SE*	*t*
Age	-0.11	0.09	-1.25	-0.17	0.09	-1.95	-0.22	0.08	-2.71**
Resilience	-0.42	0.09	-4.86	0.33	0.09	3.75***	-0.45	0.09	-4.98
Anterior insula							-0.03	0.09	-0.39
OFC-TP							-0.19	0.08	-2.23*
	R^2	0.20			0.13			0.33	
	F	13.60***			8.14***			13.72***	

Note: OFC-TP: the functional connection strength between left OFC and right TP; SE: standard error. *β:* standardized regression coefficient; $^{*}p < 0.05$, $^{**}p < 0.01$, $^{***}p < 0.001$.

Table S9. Paths and effect size for parallel mediation model using anxiety symptoms as the dependent variable

	Effect size	Boot-LLCI	Boot-ULCI
Direct effect			
**resilience→anxiety**	**-0.448**	**-0.626**	**-0.270**
Indirect effect			
resilience→anterior insula→anxiety	0.014	-0.071	0.117
**resilience→OFC-TP→anxiety**	**-0.062**	**-0.131**	**-0.012**
**Total effect**	**-0.496**	**-0.654**	**-0.338**

Note: Bold parts indicate significant paths; LLCI = lower level of 95% confidence interval, ULCI- upper level of 95% confidence interval. OFC-TP: the functional connection strength between left OFC and right TP.

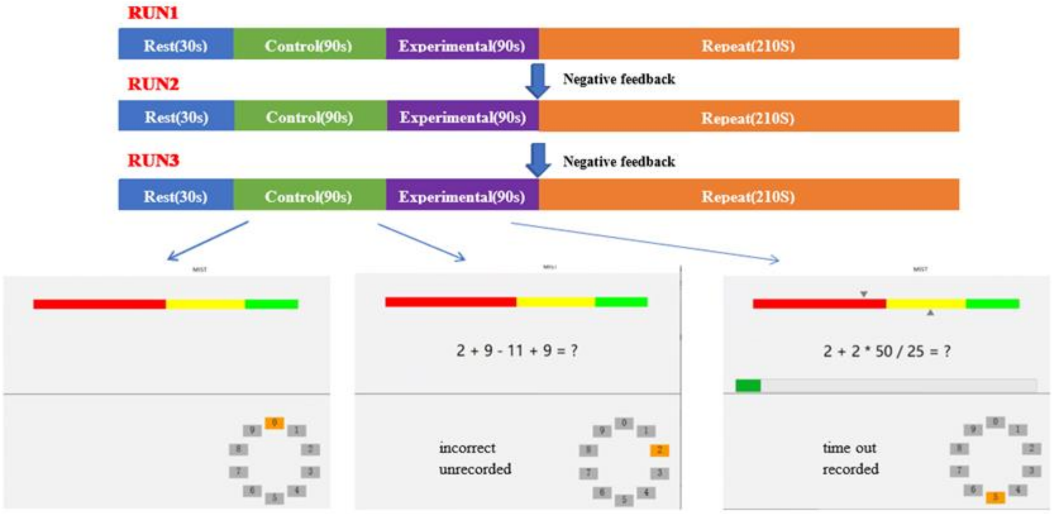

Figure S1. An overview of the Montreal Imaging Stress Task. The colored bar at the top of the screen represents the number of correct responses by the subject (top arrow = average performance, bottom arrow = current subject's performance)

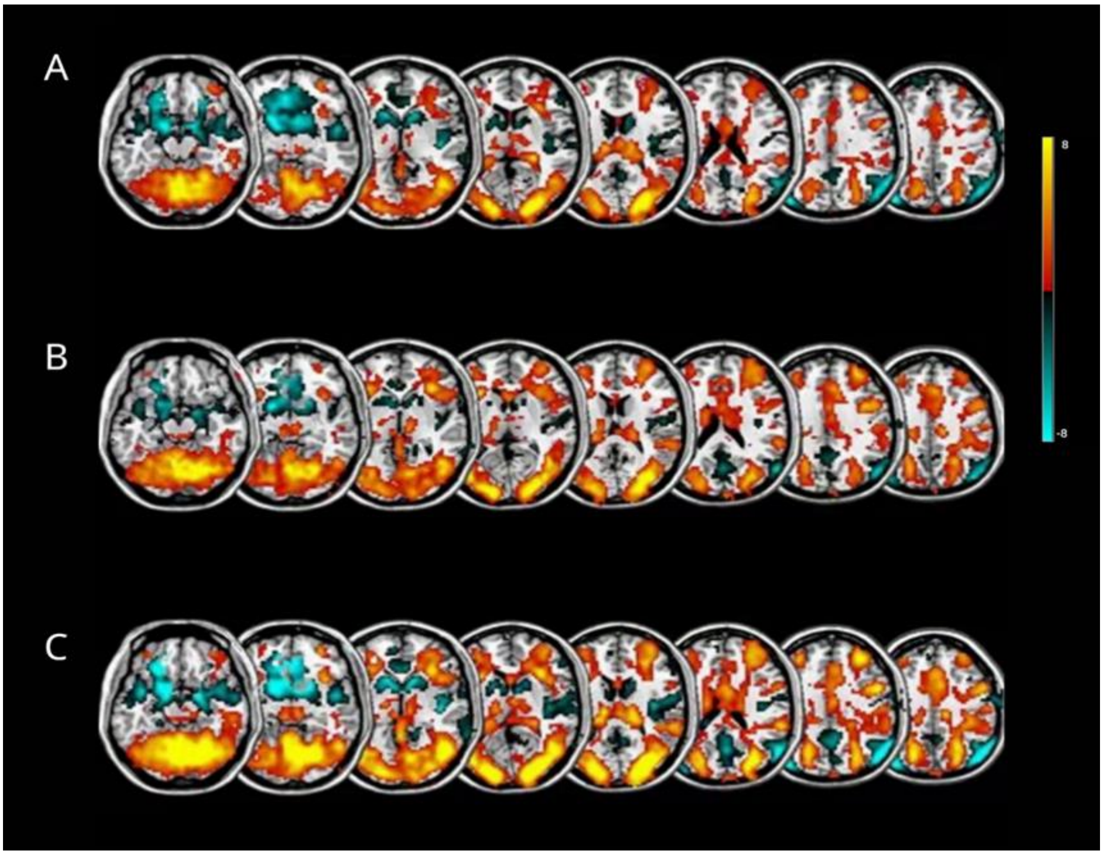

Figure S2 Brain functional activation/deactivation induced by stressful tasks. (A) all subjects; (B) high resilience group; (C) low resilience group. Red indicates areas of brain function activation, and blue indicates areas of brain function deactivation ($p_{\text{FWE-voxel}}$<0.05).

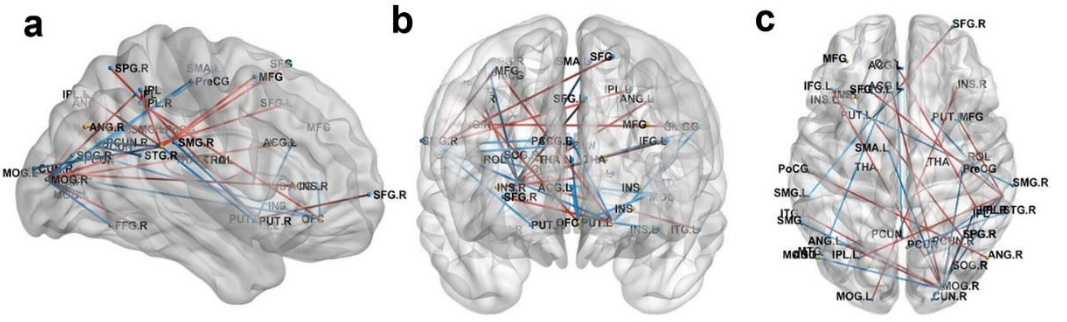

Figure S3 The brain functional connection patterns of the whole group under stress. a: Sagittal, b: Coronal, c: Axial. The yellow points mean the seed points, and the blue points are the whole-brain connection results of the seed points. The red line means the functional connectivity between the two brain regions is significantly enhanced, and the blue line means the functional connectivity between the two brain regions is significantly weakened ($p_{\text{uncorrected-voxel}} < 0.001$, $p_{\text{FWE-cluster}} < 0.001$). ANG- Angular; INS- Insula; MOG- Middle occipital gyrus; PUT- Putamen; SFG -Superior frontal gyrus; SPG-Superior parietal gyrus; ACG-Anterior cingulate and paracingulate gyri; SMG- Supramarginal gyrus； SFG-Superior frontal gyrus; IPL-Inferior parietal gyrus; CUN- Cuneus; PCUN- Precuneus; STG-Superior temporal gyrus; OFC- orbitofrontal cortex; IFG-Inferior frontal gyrus; SFG-Superior frontal gyrus; ITG-Inferior temporal gyrus; SOG-Superior occipital gyrus; SMA-Supplementary motor area; FFG- fusiform; L/R: Left/Right.

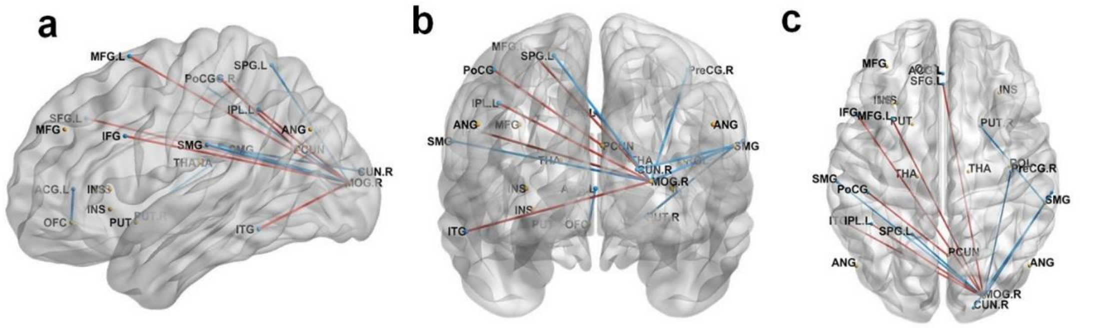

Figure S4 Brain functional connectivity patterns of the high resilience group. a: Sagittal, b: Coronal, c: Axial. The yellow points mean the seed points, and the blue points are the whole-brain connection results of the seed points. The red line means the functional connectivity between the two brain regions is significantly enhanced, and the blue line means the functional connectivity between the two brain regions is significantly weakened ($p_{\text{uncorrected-voxel}} < 0.001$, $p_{\text{FWE-cluster}} < 0.001$). OFC- orbitofrontal cortex; ACG- Anterior cingulate and paracingulate gyri; PCUN-Precuneus; PoCG-Postcentral gyrus; PUT-Putamen; CUN-Cuneus; MOG-Middle occipital gyrus; IFG-Inferior frontal gyrus; ITG-Inferior temporal gyrus; IPL-Inferior parietal gyrus; MFG- Middle frontal gyrus; SFG-Superior frontal gyrus; PreCG-Precental gyrus; SPG-Superior parietal gyrus; SMG- Supramarginal gyrus; L/R: Left/Right.

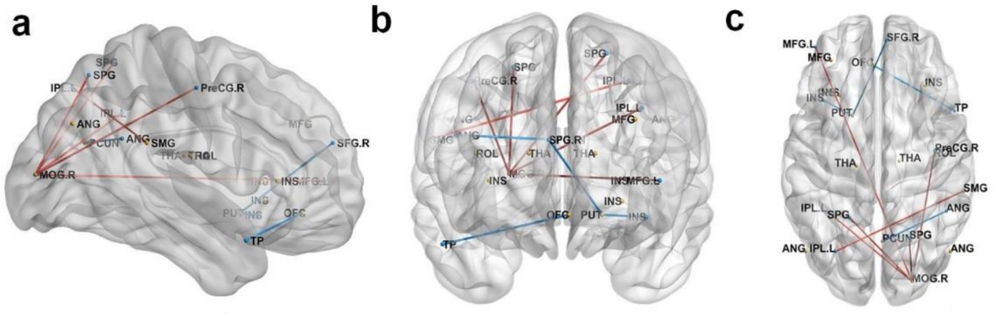

Figure S5 Brain functional connectivity patterns of low resilience group.

a: Sagittal, b: Coronal, c: Axial. The yellow points mean the seed points, and the blue points are the whole-brain connection results of the seed points. The red line means the functional connectivity between the two brain regions is significantly enhanced, and the blue line means the functional connectivity between the two brain regions is significantly weakened ($p_{FWE\text{-}cluster} < 0.001$). PUT-Putamen; INS-insula; OFC- orbitofrontal cortex; TP- Temporal pole; PCUN- Precuneus; ANG- Angular; SMG-Supramarginal gyrus; IPL-Inferior parietal gyrus; MOG- Middle occipital gyrus; MFG- Middle frontal gyrus; PreCG-Precental gyrus; SPG- Superior parietal gyrus; L/R: Left/Right.

Reference:

Das, M. K., Chaudhary, A., Bryan, A., Wener, M. H., & Morishima, C. (2020). Rapid Screening Evaluation of SARS-CoV-2 IgG Assays Using Z-Scores to Standardize Results. *Emerging Infectious Diseases*, *26*(10). https://doi.org/10.3201/eid2610.202632

Hayes,A.F. & Preacher, K. J. (2010). Mediation and the Estimation of Indirect Effects in Political Communication Research Andrew F. Hayes, The Ohio State University, Kristopher J. Preacher, University of Kansas, and Teresa A. Myers, The Ohio State University. In *Sourcebook for Political Communication Research* (434–465). Routledge.

Pandey, D., & Shrivastava, P. (2017). Mediation effect of social support on the association between hardiness and immune response. *Asian Journal of Psychiatry*, *26*, 52–55. https://doi.org/10.1016/j.ajp.2017.01.022

Podsakoff, P. M., & Organ, D. W. (1986). Self-Reports in Organizational Research: Problems and Prospects. *Journal of Management*, *12*(4), 531–544. https://doi.org/10.1177/014920638601200408
